# Mobile Application to identify and recognize emotions for children with autism: A systematic review

**DOI:** 10.3389/frcha.2023.1118665

**Published:** 2023-03-13

**Authors:** Abdelrahman Mohammed Al-Saadi, Dena Al-Thani

**Affiliations:** Information and Computing Technology, College of Science and Engineering, Hamad Bin Khalifa University, Doha, Qatar

**Keywords:** autism spectrum disorder (ASD), mobile applications, emotion, children, smart phone, autistic, child, feeling

## Abstract

**Introduction:**

Emotions are a vital component of human interaction. Children with Autism Spectrum Disorder (ASD) face severe difficulties in sensing and interpreting the emotions of others, as well as responding emotionally appropriately. Developers are producing many mobile applications to assist ASD children in improving their facial expression detection and reaction abilities and increasing their independence.

**Objective:**

This systematic review aims to explore the mobile application in helping children with ASD to identify and express their feeling.

**Methods:**

The inclusion and exclusion articles for our analysis were mapped using the PRISMA Preferred Reporting Items for Systematic Reviews and Meta-Analysis diagram. The studies were retrieved from the following four databases: Google Scholar, Scopus, Association for Computing Machinery (ACM), and Institute of Electrical and Electronics Engineers (IEEE). Additionally, two screening processes were used to determine relevant literature. Reading the title and abstract was the initial step, followed by reading the complete content. Finally, the authors display the results using a narrative synthesis.

**Results:**

From four electronic databases, we retrieved 659 articles. six studies that met our inclusion criteria were included in the systematic review. More details about inclusion and exclusion criteria can be found in the Eligibility criteria.

**Conclusion:**

This systematic review sheds light on current research that employed mobile applications to improve emotion detection and expression in children with ASD. This smartphone application has the potential to empower autistic children by assisting them in expressing their emotions and enhancing their ability to recognize emotions. However, it is currently deemed essential to assess the effectiveness of mobile applications for remediation through more rigorous methodological research. For example, most included studies were quantitative and focused on statical measurements. However, there is an immediate need for more incredible research in this area to include qualitative research and to consider large samples, control groups and placebo, prolonged treatment durations, and follow-up to see whether improvements are sustainable and to ensure the effectiveness of applications.

## Introduction

The proliferation of mobile devices alters how we interact with and understand the world. Nowadays, it is not uncommon for smartphones and tablets to have facial recognition software, and wireless biosensors can assess factors like a person's alertness, focus, or even meditation. With these enhanced capacities, developers can make various emotional intelligence apps (1). Children with Autism Spectrum Disorder (ASD) are a population that would benefit significantly from emotion-aware apps. In contrast, smartphones and tablets can identify facial expressions, and wireless biosensors can assess their degrees of alertness, focus, and relaxation (1). Autism spectrum disorder (ASD) is a term used to describe symptoms that include difficulties with social communication and repetitive sensory-motor behaviors that manifest at an early age and have a significant genetic component and other factors (2). Autistic Disorder, Asperger's Disorder, and Atypical Autism are the three primary subtypes of ASD. People with Asperger's Disorder and Atypical Autism share similar symptoms and are both considered mild versions of the autistic spectrum (3). Despite cultural, racial, ethnic, and socioeconomic differences, children with ASD share essential traits in two areas: social communication and confined, repetitive sensory-motor behaviors (2). In addition, various symptoms, including cognitive, behavioral, emotional, and sensory problems, are reported. Sleeping deprivation and eating disorders, synesthesia, emotional instability, and challenges with initiating, thinking, and organizing are frequently observed in people with autism (4). Hyperactivity and attention deficits [such as attention-deficit/hyperactivity disorder (ADHD)], anxiety, depression, and seizures are pretty common co-occurring mental or neurological illnesses among people with autism, along with these core symptoms (2). On a global scale, ASD affects roughly one in every hundred children (5). ASD has a male preponderance of more than four to one occurrence worldwide (6). As many as 47% of children diagnosed with ASD in 2005 also suffered from another diagnosis; attention deficit or hyperactivity constituted the most common (30%) (7). 83% of children with ASD at age eight had a co-occurring developmental diagnosis, 16% a co-occurring neurologic condition, and 10% a co-occurring psychiatric diagnosis (8). Since co-occurring conditions/symptoms often lead to more significant impairment and increased demand for services, especially medications and emergency room visits for accidents, the quality of life of children with ASD and their families is negatively impacted (9). In this paper, we conduct a systematic review to examine the available studies on mobile applications to aid children with ASD in identifying and expressing their emotions. This study aims to assess the state of emotion recognition mobile apps for children with ASD and to identify existing tools, promising developments, and unmet needs in the field.

## Education and learning mobile application for autism

During the past few years, the use of mobile technologies in the education of autistic children has expanded considerably. These technologies are valuable for various purposes, including enhancing the learning environment. Mobile devices have significant potential to improve traditional learning by delivering words and actions in a more dynamic and efficient manner. Therefore, it is crucial to assist autistic children in becoming more self-reliant so that they may carry out routine tasks without assistance. Digital assistance, particularly mobile applications, enables autistic children to live independently (10).

Tools for improving the child's social and communicative abilities are one of the most fruitful application areas in ASD intervention (11). Multiple studies indicate that most individuals with ASD have a natural aptitude for technology and a favorable attitude toward computer-based training (12). According to studies, computer-based training is more successful for individuals with ASD than traditional education (13). Due to recent advancements in computer vision and deep learning, new avenues are currently being explored in computerized assistance and intervention systems for individuals with impairments (13). A considerable amount of work is being conducted in the computing field on serious game-based interventions for autistic children. Serious Games (SG) are computerized games and equipment with an instructional purpose beyond enjoyment (14). In recent years, numerous SG has been developed for instructional reasons for typically developing children and children with ASD. As a result, most SG created for those with ASD are meant to aid in therapy or education or to help them become better communicators. For various reasons, SG is widely accepted among children with ASD. First, the virtual environment lacks the pressure of the actual world, and they can first explore it freely and confidently. Second, autistic children take pride in their ability to use technology because of the high regard for these devices nowadays. It is commonly considered that children with ASD are visual learners. This indicates that users are more engaged when there are more images or animations in a framework or application. Therefore, an application created specifically for children with ASD should rely heavily on visual information, such as more graphics and animations, enabling the children to acquire topics effectively. Additionally, for a child with ASD, language should be straightforward and basic (15).

## Emotions and autism

Emotions are elements of our evolved heritage, where physical changes elicit the experiencing condition of emotion, and feelings are responses to cognitive processing (e.g., reasoning, memory, and attention). They are related to the motivation of an individual. In addition, some scholars define emotions as socially constructed syndromes or transient social roles. Across these various viewpoints, concepts like feeling, mood, affect, and affective response are commonly regarded as synonymous with emotion (16).

Autism spectrum disorder is associated with numerous deficiencies, significantly impacting emotional functioning. Anger, violence, self-injury, anxiety, and impulsivity are co-occurring disorders. Emotion regulation (ER) is a concept that may give scientific validity to emotional and behavioral difficulties identified in ASD. ER is typically defined as the spontaneous or intentional alteration of an individual's emotional state that facilitates adaptation or goal-directed behavior. ASD individuals may struggle to use appropriate ER methods and might respond impulsively to emotional inputs with tantrums, aggressiveness, or self-injury (17). These acts are frequently regarded as purposeful or defiant; however, they may result from deficient emotion control (17). ASD is characterized by emotional intelligence impairments. These deficiencies are associated with difficulties in comprehending, communicating, and regulating emotions, as well as in comprehending the feelings and emotions of others and displaying empathy (18).

## Learning and expressing emotions for autism

It is found that people with Autism spectrum conditions (ASC) lack emotional intelligence skills (19). These deficiencies are associated with impaired emotional awareness, expression, and regulation and impaired capacity for empathic understanding and behavior (19). Furthermore, these impairments are seen in assessments testing emotion detection from facial expressions, verbal intonation, and body language, as well as in ecological, life-like tasks requiring the integration of emotional cues from different perceptual channels in context (19). Even though some individuals with ASC establish compensation methods that enable them to detect fundamental emotional expressions and circumstances, a general weakness in recognizing more complicated emotions lingers into adulthood (19). These deficits in emotion detection are correlated with alterations in attentional, perceptual, cognitive, and neurological processes (19). As early as 30 months of age, parents of children with ASD have recognized abnormalities in their child's social development. As children with ASD prefer to minimize eye-to-eye gaze, poor social behavior may result from inappropriate eye-to-eye contact. As early as three, children with ASD have been shown to overlook or disregard notably negative facial emotions (i.e., sadness, fear, and discomfort) displayed by adults or in photographs (20). A child with autism may identify the primary emotions of happiness and sadness between the ages of five and seven but cannot immediately comprehend highly complex expressions, including anger, surprise, or fear. Children with less severe ASD may be able to express their emotions similarly to typically developing children but struggle to comprehend them well enough to describe them adequately. Children with more severe ASD display fewer emotions than typically developing children. They may appear heartless, lack empathy, or have rapid and inconsistent responses (21). Moreover, many authors show that children with autism are less competent at identifying different emotional expressions than typically developing youngsters as early as the ages of 5–7 years old, which indicates to start assessing and supporting ASD children before reaching this age (22). However, other research It suggests that children with autism close the gap to their normally developing classmates in emotion recognition and maintain quite equivalent into adolescence (22–24).

## Clinical impact statement

This evaluation sought to comprehend the existing mobile application used to test autistic children's ability to detect and express emotions. The findings indicate that only some studies employ mobile applications and games in marginalized communities. Six studies demonstrated that these applications have the potential to help children with ASD perceive and express their emotions. However, there is an urgent need for additional study in this area.

## Materials and methods

### Eligibility criteria

Initially, all titles and abstracts were evaluated to ensure that they met the following inclusion criteria: (1) the study focused on children with autism regardless to their gender and race (i.e., individual who is 18 old and older were excluded); (2) the study mainly focused on the use of mobile applications that help autistic children recognize and express their emotions, (3) the study includes at least one experiment, pilot study, or trial involving individuals with ASD (i.e., study with no participants were excluded); and (4) the study should be written in English language (i.e., study written in other language were excluded). Finally, we did not include any studies that were merely reviews, meta-analyses, book chapters, secondary research, proposals, thesis, editorials, abstracts only, or commentary. To ensure that all relevant items were included, we did not impose any time restraints. All eligibility requirements and exclusions are summarized in Box 1 below.

Box 1 **Classify of inclusion and exclusion criteria.**

Inclusion Criteria

• Peer reviewed articles (Primary studies only).
• Children with autism.
• Mobile applications used to identify and express emotion.
• The age of participants should under 18 years.
• Articles must have participants.

Exclusion Criteria

• Any article that is not in English language.
• Reviews, meta-analyses, book chapters, secondary research, proposals, thesis, editorials, abstracts only, or commentary.
• Other technologies such as Computer and laptops.

### Search process

To conduct a systematic review four databases were researched. The four databases are Google Scholar, IEEE Xplore, Association for Computing Machinery (ACM), and Scopus without any time restriction. The keywords and terms that were utilized in research are [(“Mobile app*” OR “Smart phone” OR “Mobile phone” OR “Mobile device” OR “Tablets”) AND (“Autism” OR “Autism Spectrum Disorder” OR “children with autism” OR “autistic children” OR “autistic child”) AND (“feel*” OR “emotion” OR “sentiment” OR “reaction” OR “empathy” OR “affection”)].

### Selection of the studies

**Figure 1:**
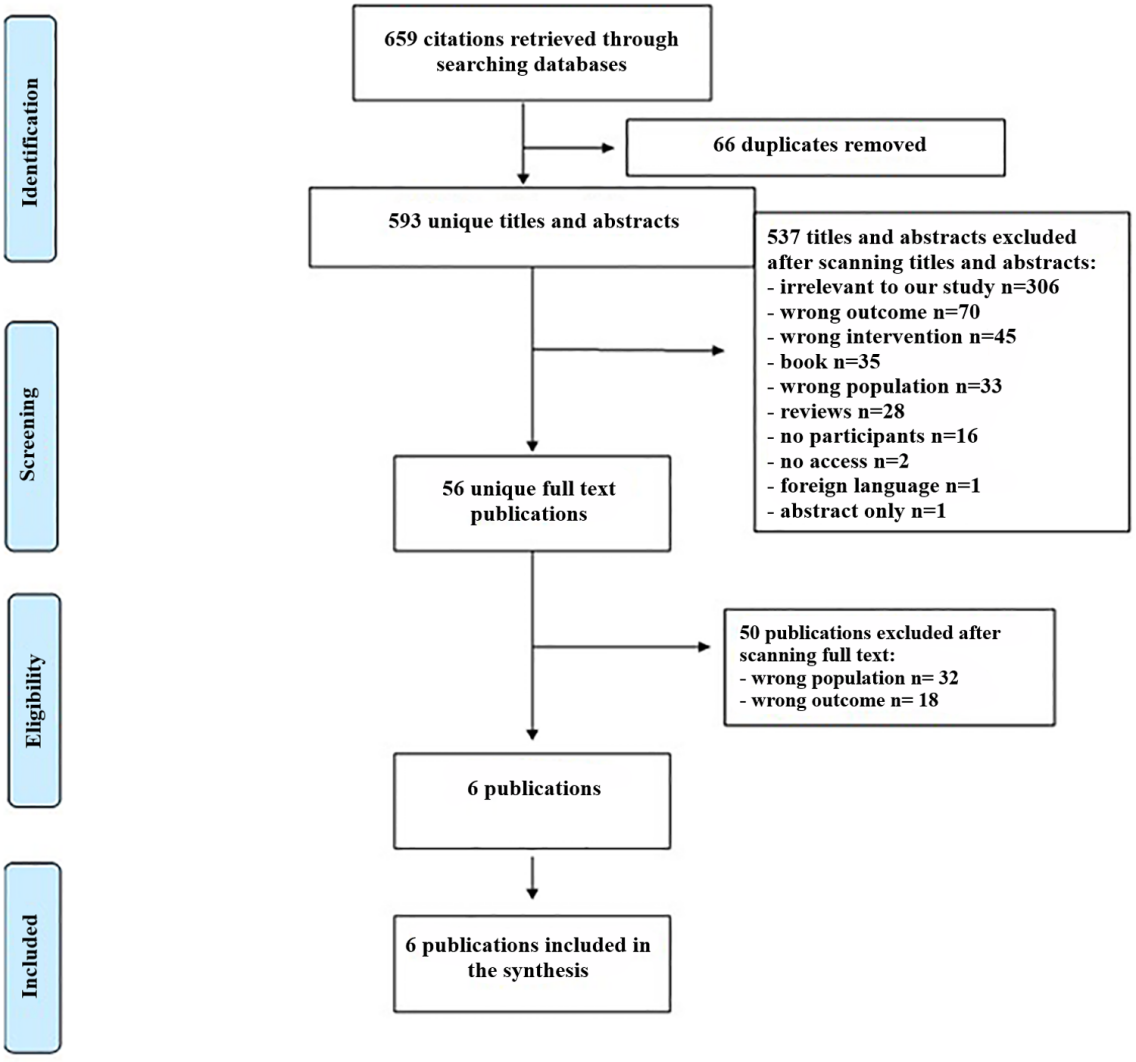
The selection process - PRISMA (Preferred Reporting Items for Systematic Reviews and Meta-Analysis).

For proper structuring of the articles, we have used the Rayyan tool to eliminate duplicates and make study selections. First author with helping of second author used Rayyan tool for selection of the studies. The study selection process consisted of two parts. Using Rayyan, Al-Saadi has examined the title and abstract to eliminate any selection bias. The second stage was to read the entire paper. Dr. Dena supervised the study selection and data extraction processes. Eventually, the dispute was addressed, and both researchers reached consensus on the studies. The selection process is shown below in Figure 1.

### Data extraction

Al-Saadi and Dr. Dena agreed to build an Excel file that summarized all study data to assist data extraction. The two researchers labored to extract data from the chosen studies. The extracted data include the publication date, the type of mobile application used, and whether the study involved artificial intelligence or machine learning techniques. Dr. Dena also improves the data extraction method by guiding the whole process. Review of the selected articles is presented below in Table 1 and the summary of selected articles are presented in Table 2.

**Table 1:** Paper types and venues of the selected studies.

References	Authors (year)	Venues	Type
(25)	Garcia-Garcia et al. (2019)	Interacción 2019: XX International Conference on Human Computer Interaction	Conf
(26)	Cheng et al. (2016)	5th IIAI International Congress on Advanced Applied Informatics	Conf
(27)	Anishchenko et al. (2017)	12th International Joint Conference on Computer Vision, Imaging and Computer Graphics Theory and Applications (VISIGRAPP 2017)	Conf
(28)	Garcia-Garcia et al. (2021)	Universal Access in the Information Society	Journal
(29)	Zhang et al. (2019)	International Journal of Developmental Disabilities	Journal
(30)	Zoerner et al. (2016)	16th International Conference on Advanced Learning Technologies	Conf

**Table 2:** Summary of selected studies (*n* = 6).

Authors (year)	Target skill	Participants	Intervention setting/Name/Time	Research design	Outcome measurement	Outcomes
Garcia-Garcia et al. (2019)	Identify and express emotions	3 participants, (8–10) years old	Mobile app Android (*EmoTEA*) T: 3–5 min	Experimental study	System Usability Scale Test (SUS test) questionnaire	Create a serious game without distracting components so kids may learn to identify and express emotions from faces.
Cheng et al. (2016)	Identify and express emotions	18 participants (12 M & 6 F) (9–13) years old	Mobile app Android (*3CFER*) T: three times	Randomized controlled	Complex-emotion (CE) scale, brief interview, and pretest-posttest	This mobile-emotion learning method promoted realizing emotions and engaged ASD individuals.
Anishchenko et al. (2017)	Identify and express emotions production by imitation	19 participants (17 M and 2 F) (6–12) years old	iPad iOS	Experimental study	Observation, test, family questions	10 of 19 kids were able to understand emotion and modify their behavior accordingly in daily life.
Garcia-Garcia et al. (2021)	Identify and express emotions	3 participants (8–10) years old	Mobile app Android (*EmoTEA*) T: 3–5 min	Experimental study	System Usability Scale Test (SUS test) questionnaire	Recognition of the system as a valuable teaching tool for emotion-related themes for children with ASD.
Zhang et al. (2019)	Cognition and academic skills; adaptability and regulation skills; social interaction skills; facial expression recognition and emotion understanding; communication skills; behavior assessment and support; and play skills.	3 participants (1 M & 2 F) (3–6) years old	Android and iOS (*Qunatiandi*) T: every day for 8 weeks	Experimental study	Baseline test, videos recording, and a maintenance stage followed by intervention termination.	All individuals improved in social acuity, especially emotion differentiating and comprehending.
Zoerner et al. (2016)	improve autistic children's social and emotional skills	15 participants (11 TD & 4 ASD) (7–12) years old	Mobile app Android (*Zirkus Empathico*) T: 100 min, once per week, for 6 weeks	Pilot study	Observations	First IT-based comprehensive and naturalistic treatment concept to develop pre and primary school children's socio-emotional ability

TD, typically developing; M, male; F, female; T, time and frequency.

## Synthesis

The researchers took a narrative synthesis approach for the systematic review. Such a synthesis provides the reviewers with an opportunity for introspection concerning the studies considered here. For instance, the study's authors will consider how autistic children have used various smartphone apps to label and communicate their emotions. It is also important to note the intervention employed in each study. How the intervention was implemented and what was found in each research.

## Results

### Search results

For this systematic review, we obtained 659 articles from four databases and selected six for data synthesis, as shown in Figure 1. After eliminating duplicate studies, 593 articles were left. During the initial screening of titles and abstracts, 537 studies were excluded (Irrelevant to our study discussing other diseases but not autism = 306 studies, wrong outcome (articles were focusing on other outcome such as counting skills) = 70 studies, wrong intervention (articles were focusing on other technologies such as personal computers) = 45 studies, book chapter = 35 studies, wrong population (the population were diagnosed with autism) = 33 studies, Reviews = 28 studies, no participants = 16 studies, no access = 2 studies, foreign language (articles were written by other languages but not English) = 1 study, and abstract only = 1 study). In the second phase, we screened the full texts of 56 articles and included 6 studies.

## Type, place, and year of publication

**Figure 2:**
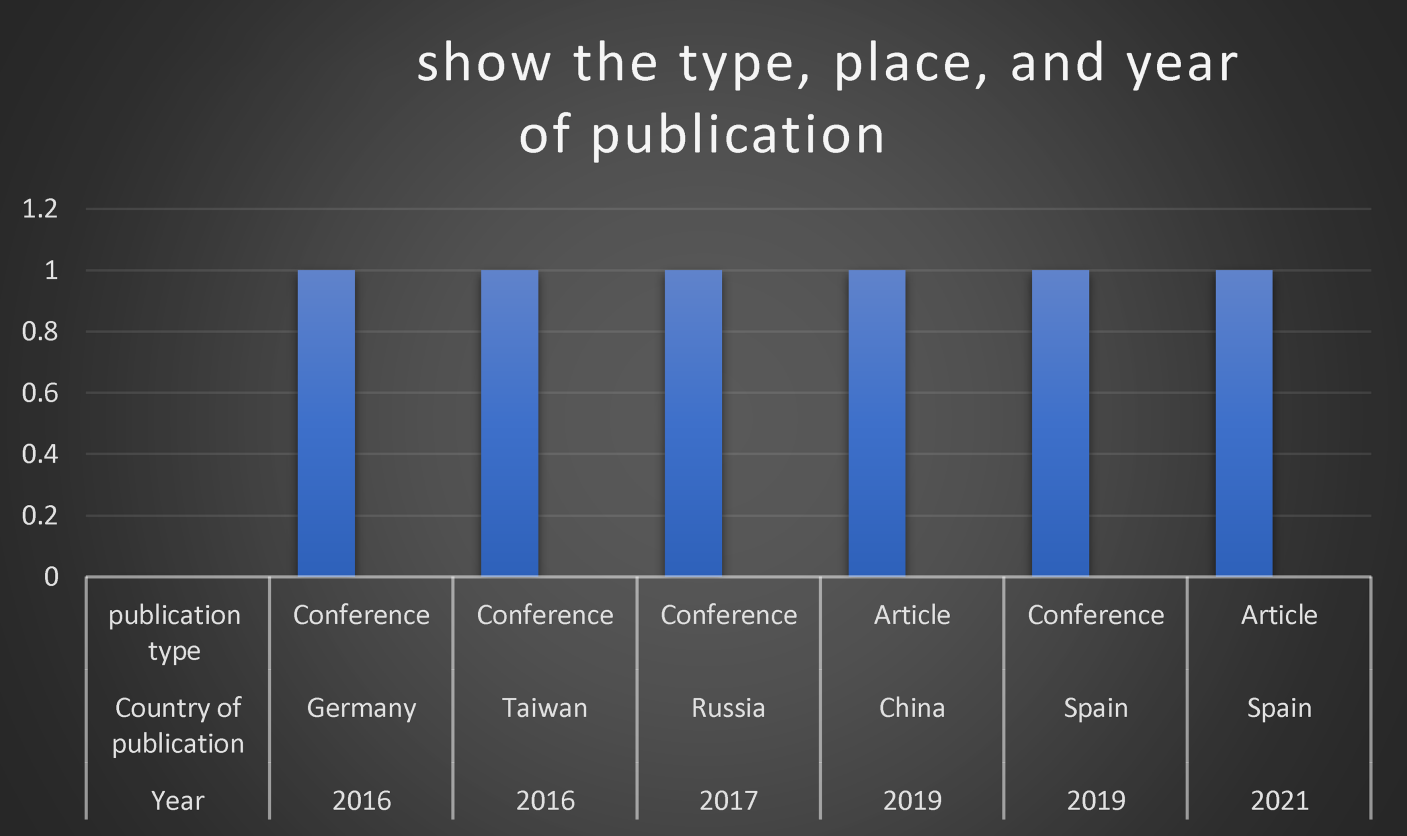
Show the type, place, and year of publication.

Studies are published in various channels based on the review outcomes. Figure 2 depicts the quantity and type of articles published each year. The majority of the included studies were published in conference (4 articles, 66.7%) and the remaining studies (2 articles, 33.3%) were published as journal articles. All included articles were released in 2016 onwards, where basically two articles (33.3%) were published in 2016 (26, 30), one study (16.7%) conducted in 2017 (27), two studies (33.3%) were published in 2019 (25, 29), and finally one study (16.7%) was released in 2021 (28). Regarding country of publication, two articles were published in Spain (25, 28), one article conducted in China (29), one study published in Germany (30), one article were released in Russia (27), and one article was issued in Taiwan (26). Concerning the design of included articles, the common design was experimental study as represented in four studies (25, 27–29) as show in Table 1. Additionally, one study used randomized controlled design (26), and the final study was designed as pilot study (30).

## Characteristics of participants

As shown in Table 2, the most significant number of participants who were included in the study was 19 participants (27), followed by 18 participants (26), and 15 participants (30). The residual four studies have only three participants (25, 28, 29). Only three papers mention the gender of their participants (26, 27, 29), and males predominated by a factor of three to one. The mean age of participants in the selected studies is 6 years and 5 months (ranging from a 3-year-old to a 13-year-old). Only one article compares the mobile application use to identify and recognize emotion between typically developed children and children with ASD (30).

## Intervention platform and target skills

Table 2 details the various mobile phones and iPads used by researchers in their studies. The vast majority of research intervention projects (66.7%, *n* = 4) are performed using the android platform, and only two studies (33.3%) were conducted on the IOS platform and android. Four articles have utilized the mobile application to enhance emotion identification, and expression skills (26–29), and one study targeted social and emotional skills (30). The last study used a smartphone application to enhance a wide range of abilities, including learning and memory; flexibility and self-control; friendship and group dynamics; reading and interpreting facial expressions and emotions; expressing oneself in words; assessing and supporting desired behaviours; and even engaging in imaginative play (29).

## Methods used to evaluate the outcomes

The findings of the selected studies were recorded using an outcome measurement approach such as questionnaires, and scaling (31). Questionnaires and surveys, scaling, qualitative studies, and unobtrusive measures are the four primary methods of measurement (31). Four main outcome assessment methods have been utilized in the selected papers, including interviews, questionnaires, observations, and scaling. Overall, multiple measuring approaches were used in 83.3% of the studies, while 16.7% relied on a lone approach (30).

## Studies outcome

As displayed in Table 2, overall, 83.3% of studies provided findings of app, framework, and conceptual design, as well as user studies (26–30). All of the included studies demonstrated an increase in participants’ capacity to identify and recognize emotion. The qualitative data from the studies (27, 30) reveals that participants developed better interpersonal and communicative competence. The qualitative results from the study (27) indicate that individuals improved their ability to recognize emotions. In addition, the tablet has the potential to substitute skilled specialists with others with less training, such as family members of children. Moreover, the researchers reported that the mobile app has the ability to enhance the instruction strategy and make it more accessible and assisted children in acquiring quite vital talents. Additionally, there were slight improvements that were noticed in the children's daily lives (29).

### Theory and approaches of mobile applications

#### EmoTEA

*Garcia-Garcia et al. (2019, 2021)*

EmoTEA is a meaningful game designed for smartphones which aimed to aid children with ASD in enhancing and growing their emotional intelligence, particularly emotional abilities surrounding emotion detection, whether those emotions are their own or those of a third party (25, 28). The game is designed for kids with ASD within the ages of 6 and 12. EmoTEA was created utilizing Tactile Interface Design and automated emotion recognition relying on facial features (25, 28). The software analyzes emotions depending on facial gestures and transforms physical things into interfaces with which the app can interact (25, 28). EmoTEA's primary objective is to address the incapacity to communicate and comprehend emotions by providing activities to recognize and articulate basic emotions (25, 28). Thus, these activities, focused on emotion recognition and mimicry, are designed to assist individuals with ASD in developing their emotional maturity (25, 28). Regardless of the flaws in Ekman's concept, the six-basic-emotions methodology has become extensively adopted in the area of Affective Computing, where it serves as the de facto categorization utilized by emotion sensors to indicate what emotions have been detected (25, 28).

#### 3D complex facial expression recognition system (3CFER)

*Cheng et al. (2016)*

The application featured a 3D humanoid with dynamic reactions and a variety of social occasions. The software material included a variety of social settings that may trigger emotion. All content was reviewed by a team of ASD specialists to ensure its appropriateness for children with ASD (26). In addition, the software could generate audio cues of the inquiry to assist players toward a possible response, and it also urged them to discover an appropriate response (26). The 3CFER concept consisted of two phases: first, a social occasion induces an emotion, followed by a circumstance that induces an emotion (26). The 3CFER application can provide instantaneous assessment on the user's correct or incorrect responses.

*Anishchenko et al. (2017)*

The software developed for tablet PCs was designed to teach face expression recognition and interpretation. It employs a recently developed computer visual algorithm for facial expressive detection that enables estimation of whether the given gesture is suitable or not and guides clients (27). The algorithms are intended to identify unrecognized phrases or Action Unit (AU) states. The purpose of a computer recognition algorithm for a teaching resource is to determine whether the facial expression captured in a photograph reflects a certain recognized emotion (27). The program offers neutral-faced photographs that can be used as a teaching aid; a relatively natural method for estimating intensity is to examine facial point geometric movement proximity (27). The application contains of two phases. First, on the basis of Ekman and Rosenberg's theory, the program includes a collection of photographs for teaching the facial regions associated with six fundamental emotions (happy, surprise, sorrow, fear, disgust, and rage). Secondly, the program can facilitate imitation-based instruction in emotion generation. In the second phase, a snapshot containing a sample is shown on the screen, and the user is instructed to replicate the sample's facial expression before pressing the button for processing (27).

#### Qunatiandi

*Zhang et al. (2019)*

Qunatiandi is a mobile application available on Android and IOS platform which is tool that integrates several scientific proof therapy modalities approved by the National Professional Development Center on Autism Spectrum Disorder (NPDC) with in US (29).

Discrete Trial Training (DTT), Pivotal Response Treatment (PRT), SCERTS, and Positive Behavior Support (PBS) are some evidence-based treatment approaches that available in this application (29). Coaching in Qunatiandi focused on seven areas: (1) educational and social development, (2) behavioral regulation and flexibility, (3) interpersonal development, (4) the potential to read and interpret facial expressions and emotions, (5) effective communication, (6) behavior analysis and assist, and (7) the improvement of play (29). The program was created predicated on Baron-proposed Cohen's Empathizing-Systemizing Theory (E-S Theory), which stated that children with autism generally under average in intellectual and emotional sympathy and normal or maybe over normal in systemizing (29).

#### Zirkus Empathico

*Zoerner et al. (2016)*

“Zirkus Empathico” is a smartphone tool that utilizes a comprehensive and ecological approach to educate children's social and emotional skills. The application comprises of four distinct teaching materials focusing on various components of social cognitive, in addition to a feature to adapt the recently acquired behavior into everyday life (30). The first component of the application is the identification and vocalization of one's own feelings, which serves as the foundation for empathetic skills. The second and third modules concentrating on conceptual empathetic training the recognition of other people’s feelings through recorded facial gestures as well as videos displaying the emotion-triggering circumstances (30). The final module focuses on enhancing emotional awareness, as the child required to articulate their emotional responses to the emotions of others. The application facilitates the transmission of ecological video stimulus, such as facial gestures and contextual movies, into daily life. The combining of visual and auditory stimulation elicits larger emotional responses from children compared with photographs alone (30). The concept of the education application is centered on autism-specific behavioral psychotherapy concepts. The application was built to accommodate demands and intellectual abilities of the target audience, and designed to be straightforward and clear, free of extraneous details, and have aspects to keep children's interest and engagement (30).

## Discussion

Young people with autism often struggle with controlling their emotions. When autistic children experience unpleasant or overly active emotions, they often act out in destructive and aggressive ways, including hurting themselves or others. As a result of their emotional and behavioral difficulties, autistic children face several obstacles in their day-to-day lives, including difficulties with social evolution, connectivity, and academics (31). Children with autism sometimes have difficulty reading facial expressions, leading to communication styles that differ significantly from their typical peers (32). For this reason, smartphone applications are now available to aid in developing these individuals’ emotional intelligence by teaching them to recognize and control their emotions (18). In light of the findings, it is anticipated that research into the use of mobile application technology for recognizing and expressing emotion in autism will rise significantly in the future years.

According to the existing research, mobile applications play a crucial role in aiding autistic children in identifying and expressing their emotions and fostering their independence. A typical issue concerning the population is the limited sample size, which may be attributable to the difficulty recruiting participants. Additional research could recruit more participants and decrease gender bias among participants. In addition, the restricted participation of parents in the studied research may result from privacy and safety concerns. Each research demonstrates a positive outcome concerning the required capabilities. From the perspective of training evaluation methodologies, studies employing survey and qualitative research measurement methods demonstrate enhanced various performances and the enhancement of participants’ capacities, whereas studies employing scale claim a growth in participants’ scores.

As far as the research setting is concerned, most research is carried out at a rehabilitation facility and private school, but one study was undertaken in a home environment (30). Training in a controlled environment may enhance the emotional abilities of autistic children to a certain amount. However, they must continue to practice detecting and expressing their emotions in authentic social settings. The most noticeable characteristic of mobile applications is their portability. The relevant study should be validated with a broader range of individuals and in a variety of settings, not just in controlled settings. Future studies can incorporate more family interventions, increase parental participation, and encourage children to lead an active lifestyle.

In contrast to the fact that none of the included research acknowledged or featured parents or their mentors, more family contexts can encourage and empower parents to engage in research. For potential future approaches, lengthening the research period is a potential solution to the problem of a short intervention time. Long-term studies on the effects of interventions using mobile apps for autism help establish such tools’ reliability and make a case for their widespread use.

Regarding application design, most currently available smartphone apps for children with ASD are deemed impractical due to a lack of necessary features such as gamification and customization plans. To increase user experience and promote mobile's widespread adoption, designers must concentrate on consumers and create interactive and high-quality products that match user demands. The designers may create a range of modes and iterations that will appeal to youngsters of various ages with autism. To keep things straightforward, depending on the needs of autistic children, app designers should create multiple versions of the same program. For instance, one version can be tailored to attract pre-schoolers, while another can target nonverbal users. The software should be developed by a multidisciplinary team, collaborating with autistic children and their parents to consider every angle. In addition, the designer can incorporate features that enable data analysis or visualization tools that demonstrate the improvement and growth of the child's skills so that they can be monitored easily.

## Limitation

We can outline the most significant issues and limits encountered by the studies we analysed as the following. The first restriction is that long-term monitoring of a child's behaviour is necessary to determine whether or not an intervention is effective. Only a single study lasted up to 2 months. Secondly, it is hard to recruit participants, which is reflected in the small sample sizes of the existing studies. The most extensive study with participants had just 19 participants (27). Research initiatives also have to focus on measurement strategies. Observers in quantitative and qualitative research methodologies must be appropriately trained to avoid increasing the error rate and decreasing the usefulness and reliability of the measurement. Research methodology, sample size, and length of intervention all may have a role in identifying mobile's most significant shortcomings in assessing children with an autism spectrum disorder. Currently, only one article in this review uses a randomized experiment as its research methodology; thus, more work needs to be done to determine whether or not this method is effective.

As with any other review, this research has several limitations, such as the utilized keywords. Add to that the inclusion and exclusion criteria we used, which may impact the number of selected primary papers. Additionally, more databases can be added to expand the pool of potential research articles. This analysis aimed to get insight into the existing mobile app used to gauge emotional understanding in autistic children. Research on the efficacy of mobile applications designed to aid autistic children in expressing and identifying emotions is mostly under-researched and shrouded in secrecy.

## Conclusion

The use of smartphones and other technological aids has been included in programs designed to improve communication skills for people with ASD. This paper provides an overview of the potential of mobile apps to aid autistic children with recognizing and articulating their emotions. This evaluation sought to comprehend the existing mobile application used to test autistic children's ability to detect and express emotions. The findings indicate that few studies employ mobile applications and games in marginalized communities. Six studies demonstrated that these applications have the potential to help children with ASD perceive and express their emotions. However, there is an urgent need for additional study in this area. Researchers are encouraged to provide context for their findings by discussing how they relate to prior research and current assumptions. Results and their potential applications need to be addressed in the broadest possible terms. Possible future study focuses could also be indicated. It is intended that this review will assist researchers, educators, and people interested in ASD with helpful information and recommendations.

## Data Availability

The original contributions presented in the study are included in the article/Supplementary Material, further inquiries can be directed to the corresponding author.
